# Obtaining patients’ medical history using a digital device prior to consultation in primary care: study protocol for a usability and validity study

**DOI:** 10.1186/s12911-022-01928-0

**Published:** 2022-07-19

**Authors:** Klara Albrink, Carla Joos, Dominik Schröder, Frank Müller, Eva Hummers, Eva Maria Noack

**Affiliations:** Department of General Practice, University Medical Center Göttingen, Georg-August-University of Göttingen, Humboldtallee 38, 37073 Göttingen, Germany

**Keywords:** App, Digital medical history taking, Primary care, User friendliness

## Abstract

**Background:**

With the help of digital tools patients’ medical histories can be collected quickly and transferred into their electronic medical records. This information can facilitate treatment planning, reduce documentation work, and improve care. However, it is still unclear whether the information collected from patients in this way is reliable. In this study, we assess the accuracy of the information collected by patients using an app for medical history taking by comparing it with the information collected in a face-to-face medical interview. We also study the app’s usability from the patients’ point of view and analysing usage data.

**Methods:**

We developed a software application (app) for symptom-oriented medical history taking specialized for general practice. Medical history taking will take place involving patients with acute somatic or psychological complaints (1) using the app and (2) verbally with trained study staff. To assess the perceived usability, patients will complete a questionnaire for the System Usability Scale. We will collect sociodemographic data, information about media use and health literacy, and app usage data.

**Discussion:**

Digital tools offer the opportunity to improve patient care. However, it is not self-evident that the medical history taken by digital tools corresponds to the medical history that would be taken in an interview. If simply due to a design flaw patients answer questions about signs and symptoms that indicate possible serious underlying conditions ‘wrong’, this could have severe consequences. By additionally assessing the app’s usability as perceived by a diverse group of patients, potential weaknesses in content, design and navigation can be identified and subsequently improved. This is essential in order to ensure that the app meets the need of different groups of patients.

*Trial registration* German Clinical Trials Register DRKS00026659, registered Nov 03 2021. World Health Organization Trial Registration Data Set, https://trialsearch.who.int/Trial2.aspx? TrialID = DRKS00026659.

## Background

A consultation in primary care takes on average 7.6 min in Germany [1]. During this time, physicians take the patient’s history of present illness, perform the physical examination and suggest and explain possible therapies. The collection of a thorough individual medical history provides the most reliable indication for diagnosis in primary care [2, 3]. However, time pressure in medical consultations often leads to misconceptions about patients’ needs and potentially avoidable misdiagnosis. Some practices use paper questionnaires to collect specific information prior to consultation, especially from patients who are new to the practice. Such questionnaires can include acute medical history, allergies, drug intolerances, or previous treatments, and are neither standardized nor validated. That way, information can be collected comprehensively and expeditiously, but to be of use in the long term, it needs to be transferred into patients’ electronic medical records (EMR). This procedure is tedious for the practice staff. If done by scanning the questionnaire, the resulting document is an attachment to the EMR and of limited use. Manual transcription of the data into the EMR is even more time consuming and prone to mistakes.

Digital tools designed to collect patients’ medical history before consultation could streamline this process and improve quality of care by helping medical professionals to collect a thorough medical history. Their use might augment their work efficiency by reducing documentation work. Additionally, as information is already available to health care providers before the consultation, they can use this data for better preparation, treatment planning and even triage. There are several tools that were developed for medical history taking from specific patient populations, e.g. cardiology [4] or gastroenterology [5, 6], but only few are designed for use in general practice, or out-of-hours primary care [7, 8]. Although these tools are getting more and more common, it still remains unclear if the information collected is valid and thus can be relied on. As a basic prerequisite for the valid collection of information, the usability for different user groups (e.g. different age, sex, health literacy) must be guaranteed. Usability of such tools have been demonstrated among some smaller cohorts [9], but necessary research findings especially among certain subgroups are still lacking.

In the project “DASI” (acronym for ‘Digitally assisted system to obtain patients’ medical history before consultation’), we have developed a software application (app) for structured medical history taking for primary health care. It enables to take a symptom-oriented medical history based on the 39 most frequent complaints in general practice.

### The aim of the study

In this study we want to (1) assess if patient data obtained by the DASI-app corresponds to the information collected in a medical interview (concurrent validity) and we aim to (2) study the app’s usability as perceived by patients and analysing usage data. For both aspects, validity and usability, will assess the impact of sociodemographic characteristics.

## Methods

### Used software

**Fig. 1:**
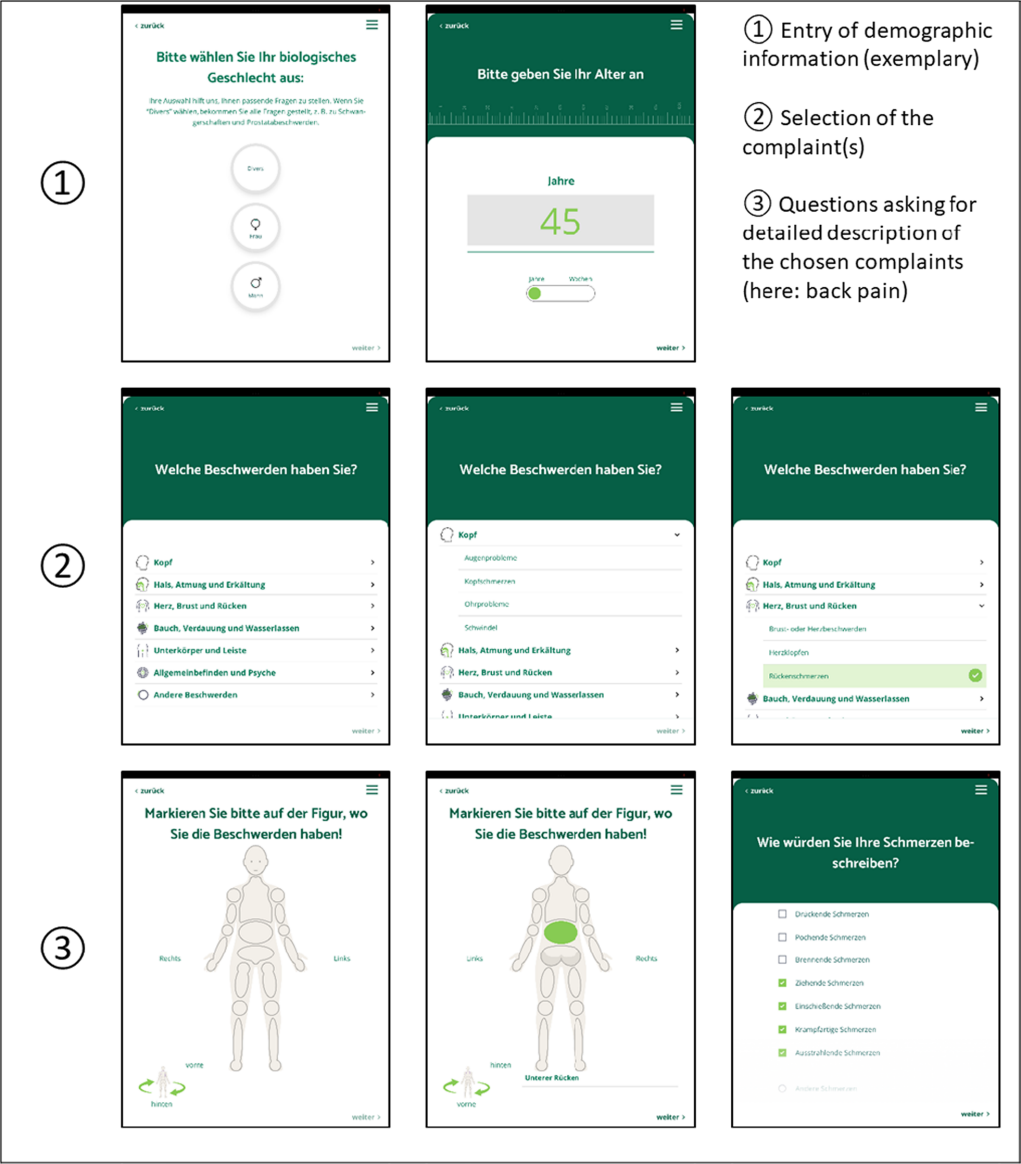
Screenshots of the app (iPad mini). (The figure was created with PowerPoint by Microsoft Office Professional Plus 2016)

**Fig. 2:**
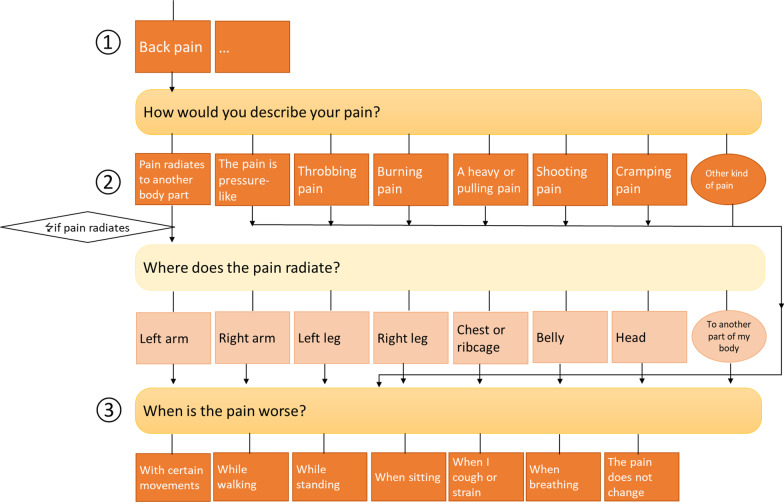
Examples of independent questions and questions triggered by former answers using the example of the complaint ‘back pain’ ①.② Patients who specify the pain as ‘radiating’ are asked to which region of the body the pain radiates to. ③ The question about the intensity of pain during certain activities is again independent of the previous questions. (The figure was created with PowerPoint by Microsoft Office Professional Plus 2016

We use a self-developed app that is designed to take the history of present illness from patients in general practice and out-of-hour practices (Fig. 1 shows exemplary screenshots). The app is based on a previous version that was used to collect medical history data from non-German-speaking patients in an initial reception facility for refugees and asylum seekers [7, 10]. Based on guidelines and health literature and tested by experienced general practitioners, the app asks patients about their present illness, including its subjectively perceived severity. Content and structure were adapted and refined for primary care by the aidminutes GmbH together with the Department of General Practice at the University Medical Center Göttingen. At the beginning, general information such as sex, height, weight and age are taken from all patients. Subsequently, the patients select one or several symptoms that as the reason for the appointment and are asked symptom-related questions. Patients’ responses trigger further specific questions about the chosen complaints (cp. Fig. 2), e.g. how and when they have started. If relevant, patients are asked about pre-existing conditions, previous treatments and surgeries, current medication, and living habits, such as drinking and smoking, as well as diseases in the family. Questions and answers are phrased in plain language. Single- and multi-selection questions are to be answered by ticking checkboxes; age, height and weight is entered using the keyboard. The design is kept simple. Menu navigation should be intuitive, so that the app can be used without prior instruction by the patients, e.g. in the waiting room before to seeing a healthcare provider. The DASI-app is designed for general practitioners as well as in out-of-hour practices. General practitioners are the first port of call for any type of medical concern. General practice is aimed at a long-term provision of health care. Out-of-hour practices provide urgent medical care for acute but not life-threatening cases when other practices are closed. The aim of the app is to provide health care providers a comprehensive summary of the current medical history in the EMR before seeing a patient. Thus, doctors are better prepared for the upcoming consultation and can directly and eventually better address patients’ health needs.

For this study, the DASI-app will run on iPad mini device, but it can run on any device with an internet browser.

### Validation

We aim to evaluate whether the app collects the patient’s current medical history as if it had been obtained in a medical consultation in an interview situation (gold standard). Therefore, we will compare the data generated when patients are using the app independently with the data collected in a medical interview which follows the same thematic structure as the app.

If the answers given in the app and verbally do not match, this may change the rest of the question sequence or leave the rest of the question sequence unaffected. In both cases, it is possible to evaluate whether the non-match is relevant for the history of present illness. For example, if questions concerning signs and symptoms that suggest a potentially serious underlying disease are answered differently.

All patients answer standardized questions including patient characteristics and complaints which were the reason for the appointment. These initial answers can be compared in all patients. Further questions on separate complaints can be compared in participants who stated the same complaints in both time points. Data will be analyzed using contingency tables. Accuracy (with its confidence interval), sensitivity and specificity will be computed in yes–no questions. For contingency tables exceeding 2 × 2 only accuracy and p value of the McNemar-test will be reported. Besides, we will analyse subgroups e.g. by age, health literacy, complaints.

### Evaluation of usability

Patients will be asked to evaluate the usability of the app with the System Usability Scale (SUS) [11, 12] which scores 10 statements on five-point-Likert scales [13]. We will use an adapted version of the SUS in German language.

Furthermore, patients will be asked about their daily use of other technological devices, whether German is their native language, and questions assessing their health literacy, reflecting the understanding with which patients navigate in the medical jargon [14] 15.

SUS scores will be compared with published norm data [16]. Further sociodemographic factors (e.g. age, native language German or not), media usage and health literacy will be explored as possible associated factors with perceived app usability, e.g. using linear regressions.

### Data collection

Data collection will take place in a convenience sample of urban and rural primary care and out-of-hour practices in Germany. Potential study participants are patients with acute somatic or/and psychological symptoms.

#### Inclusion criteria for patients

Patients who meet the following criteria are eligible to participate in the study:

- Seeking care in a participating practice
- At least 18 years old
- Consenting to participate in the study

#### Exclusion criteria for patients

Patients who meet the following criteria cannot participate in the study:

- Younger than 18 years old (legally minor)
- Patients in an apparent emergency situation
- Patients who require immediate medical treatment
- Patients who are unable to consent.

**Fig. 3:**
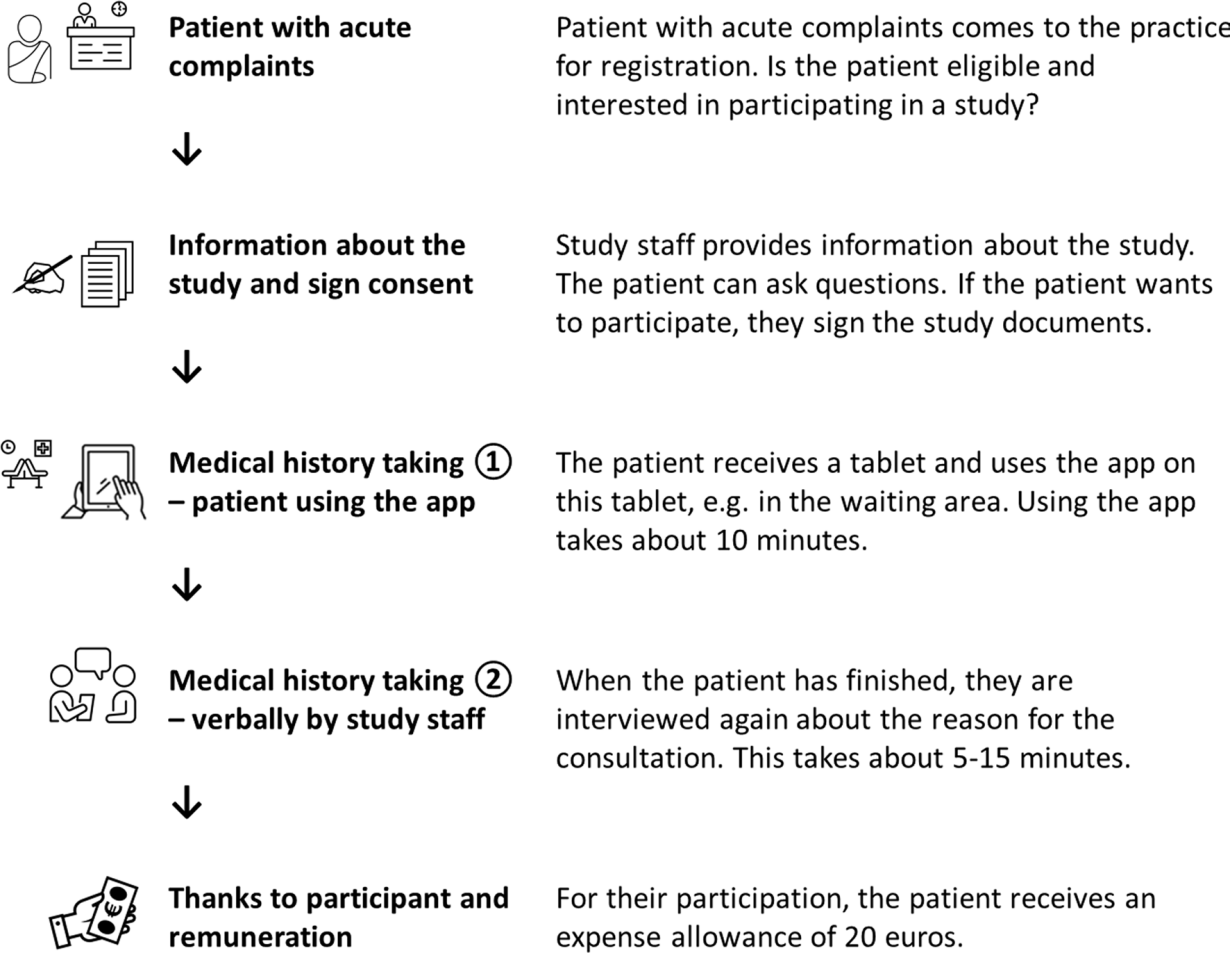
Course of the data collection. (The figure was created with PowerPoint by Microsoft Office Professional Plus 2016, partly using icons from Noun Project (https://thenounproject.com; patient by Minh DoVN, Reception by ibrandify PK, waiting room by Aficons ID, iPad by Andrey Vasiliev RU, Interview by Cuputo ID, euro payment by b farias CL.)

Participating in the study and using the app is voluntary. Patients will be addressed in the waiting situation preceding the doctor’s consultation (Fig. 3). After verifying the inclusion and exclusion criteria, the study staff will inform the patients about the study, including data protection regulations. Written informed consent and the signed privacy policy are obtained from participating patients. Patients who do not wish to participate or who cannot participate because of exclusion criteria will be registered in screening lists that include sex, birth year (requested from the patients) and the reason for non-participation (exclusion criterion, refusal or other). If patients choose to participate, the study staff will hand out a tablet on which the app is installed, asking patients to enter their medical history. The patients will use the app independently in the waiting room. The study staff will be present to help only if questions occur. Depending on the patients’ complaints and past medical history this is expected to take about 10 min. Subsequently, the patients will answer the questions of the SUS, as well as questions regarding their media use, health literacy and native language. After finishing the survey and returning the tablet, trained study staff will obtain the current medical history verbally in a separate room. The asked questions are following the app’s routine, and answers are also documented using the app. It should be noted that medical history taking by using the app’s items is probably more comprehensive than in routine medical interview. If patients give additional information, which do not appear in the app, these will be annotated. Personal information allowing patient identification is not collected by the app, data from the app and oral medical history will be linked by paired anonymous codes. Each participant will be assigned two identification numbers that belong together. The Study staff is blinded towards the content entered previously into the app by the patients. After completing the study, patients receive 20 Euros as a compensation for their participation. Afterward, the patients will go back to the waiting area and can proceed to the doctor’s consultation. There will only be one study nurse in a practice at a time. Once a patient has finished participation the study staff will address the next patient.

### Sample size

We aim to include 350–400 patients within a frame of about 12 weeks. Since some of the selectable complaints are rare, a sufficiently large number of study participants is needed to form subgroups that are large enough.

### Challenges

Within the SARS-CoV-2 pandemic it may be difficult to find general practitioners, who agree on opening their practices to study staff and are able to provide a separate room. Many practices are currently trying to minimize the number of contacts in their waiting areas. Patients with signs of an infection are now often called in at specific times or advised only by telephone or video. These patients might be underrepresented when compared to non-pandemic circumstances and may lead to a selection bias. Additionally, under-recruitment may occur and an extension of the recruitment period might be necessary.

## Discussion

The DASI project aims to improve patient care using the advantages of digital tools. In 2020, only 5% of primary care physicians in Germany used digital medical history forms, nearly 30% stated that they would like to have the opportunity to do so [17]. The reason may be that the introduction of immature digital applications with insufficient benefits and/or high hurdles for utilization has frustrated practice employees in the past. In order not to jeopardize the acceptance of digitalization there is a need for attractive digital applications that provide a practical implementation for the practices [18]. Although there are similar software developments [8, 9, 19–21], DASI is the first app for medical history taking for the use in general practice that is developed in close and independent scientific supervision.

It is not self-evident that the medical history taken by digital tools is ‘true’ and corresponds to the medical history that would be taken verbally. This, however, may not be the case, e.g. for mental or shame-related complaints, for rare (combinations of) complaints, where the predefined items do not fit, or for different user groups. Still, to date, no respective research has been conducted. It may be that patients find it easier to confide in an ‘impersonal’ tool than in a person, but the opposite could also be true. Therefore, it is important to learn whether and how patients answer questions about their medical conditions and medical history differently in a digital tool than in a face-to-face interview. If simply due to a design flaw patients answer questions about signs and symptoms that indicate possible serious underlying conditions ‘wrong’, this could have severe consequences. At the same time, digital tools for medical history taking offer the opportunity to better address patients’ needs and to improve quality care. This may be especially the case in out-of-hour practices. These are staffed with doctors of various specialities and there is little time to identify serious health problems. Besides, patients and care providers usually do not know each other. These aspects make medical history taking even more challenging than in general practice where in long-lasting doctor-patient relationships, practitioners know large part of their patients’ histories.

The validation of the app-based medical history lays the foundation for the next steps in the app development, the creation of a synopsis of patients’ complaints and multilingual use. The evaluation of the usability and the additional feedback given by study participants offers the opportunity to improve the app, to ensure that the app meets the need of the users and therefore increase the likelihood of long-term implementation. In this study, we focus on the validation and usability of the DASI-app. After studying these important aspects and incorporating respective improvements, the next step is to make it usable for physicians and practice staff. Questions and responses are designed in such a way that a concise but comprehensive synopsis can be produced, which reflects the content of the ‘conversation’ and can be understood without knowing what has been asked. This will facilitate to integrate the information to the electronic health records and permits to investigate the app in everyday practice. Here, it should be noted that, even if the DASI-app proves to be usable for patients and serves to collect valid information for physicians, once implemented to practice it needs to be studied if it also improves patient care (e.g. by increasing diagnostic accuracy or patient satisfaction or by positively influencing patient outcome).

## Data Availability

The datasets used and analysed during the study are available from the authors upon reasonable request, under consideration of the existing ethics committee vote and the legal framework conditions.

## Abbreviations

App: Software application
EMR: Electronic medical records
DASI: Digitally assisted system to obtain patient’s medical history before consultation
